# Application Value of Rehabilitation Nursing in Patients with Stroke Based on the Theory of Interactive Standard: A Randomized Controlled Study

**DOI:** 10.1155/2021/9452765

**Published:** 2021-10-26

**Authors:** Ningning Li, Jun Wang, Mei Zheng, Qunying Ge

**Affiliations:** ^1^Department of Neurology, Jiaozhou People's Hospital, Jiaozhou, China; ^2^Department of Medical Imaging, Qingdao Fifth People's Hospital, Qingdao, China; ^3^Geriatric Department, Qingdao Fifth People's Hospital, Qingdao, China; ^4^Hemodialysis Room, Jiaozhou People's Hospital, Jiaozhou, China

## Abstract

**Objective:**

To explore the application value of rehabilitation nursing based on the theory of interactive standards in stroke patients.

**Methods:**

A total of 120 stroke patients who were treated in our hospital from December 2018 to September 2020 were selected as the research objects, and the patients were divided into a control group (60 cases) and an observation group (60 cases) according to the random number table method. The control group used routine nursing care, and the observation group used interactive rehabilitation care based on the control group. The Barthel Index, National Institute of Health Stroke Scale (NIHSS) score, Specific Quality Of Life Scale (SS-QOL) score, rehabilitation standard rate, nursing satisfaction, improvement time of limb function, and compliance with rehabilitation exercise were compared between the two groups of patients.

**Results:**

After intervention, the Barthel Index of the two groups increased, and the Barthel Index of the observation group was comparatively higher (*P* < 0.05); the NIHSS scores of the two groups of patients reduced, and the NIHSS scores of the observation group were significantly lower than those of the control group (*P* < 0.05); the SS-QOL scores of the two groups of patients improved, and the increase in SS-QOL scores in the observation group was found to be significantly higher than those in the control group (*P* < 0.05); the compliance rate was found to be in favor of the observation group (83.33 (50/60) vs 63.33 (38/60)) (*χ*^2^ = 6.136, *P*˂0.05); the total satisfaction of nursing care of patients in the observation group was superior to the control group (96.67% vs 78.33%) (*χ*^2^ = 9.219, *P*˂0.05); the limb function improvement time of the observation group was significantly shorter (*P* < 0.05); the observation group had significantly higher rehabilitation exercise compliance scores (*P* < 0.05).

**Conclusion:**

The rehabilitation nursing based on the interactive standard theory can promote the stroke patients to complete the rehabilitation goals, improve the neurological and limb functions, and enhance the patients' daily living ability, quality of life, and nursing satisfaction, which is worthy of clinical promotion and application.

## 1. Introduction

Stroke, also known as the cerebrovascular event, is a far commoner acute cerebrovascular disease. Universally, it is a disease of brain tissue damage caused by sudden rupture of cerebral blood vessels or under the condition that blood cannot flow into the brain normally due to blockage of blood vessels. The high prevalence and incidence remain the major cause of morbidity and mortality in cerebrovascular disease. Sequelae such as hemiplegia generally arise after survival, and simultaneously, the patient's limb function, living ability, quality of life, etc. are compromised [1]. The decline in the ability of daily living activities leads to psychological pessimism and loss of confidence in themselves, family, and society, resulting in poststroke depression, anxiety, and other emotional changes, and these changes have a greater impact on the prognosis. Taking targeted rehabilitation nursing measures in the early stage of the disease plays a crucial role in stroke patients [2].

For rehabilitation nursing after stroke, it is advocated to start early and rehabilitation exercise can be started 48 hours after the vital signs are stable. Many literature studies have shown that the recovery of limb function is the fastest within 3 months after stroke and 90% of neurological function recovery occurs within 3 months after stroke. Early rehabilitation care is a set of standardized rehabilitation nursing models established for stroke, which is documented to speed up the recovery of stroke patients, improve limb function, boost living ability and quality of life, and reduce the occurrence of sequelae [3].

The interactive standard theory refers to that the focus of nursing is people, and the goal of nursing is to promote and maintain the health of the body. It mainly expounds the interaction between people, especially between nurses and patients, to promote the establishment of common goals and achieve the goals through the efforts of both sides [4]. The interactive standard theory emphasizes the mutual influence between individuals' interpersonal and social relationship, and nursing staff and patients jointly participate in the nursing process and interact with each other to combat diseases, so as to realize a robust outcome [5,6]. To date, scanty data are available regarding the application of rehabilitation nursing in stroke patients based on interactive standard theory and its nursing effect remains to be verified. In this regard, this study advanced the understanding of rehabilitation care in stroke patients based on interactive standard theory, and attempts were made to provide a reference for the optimization of stroke nursing.

## 2. Materials and Methods

### 2.1. Subjects

A prospective randomized controlled design was used in this study. A total of 120 stroke patients who were treated in our hospital in the time frame of December 2018 to September 2020 were enrolled and randomized to control group and observation group, 60 cases in each group. This study was approved by the Medical Ethics Committee of Jiaozhou People's Hospital (ethics number: 2017-12128) in December 2017. All patients or their family members signed informed consent.

Control group included 38 males and 22 females, with age 50–75 years and average age (64.12 ± 3.58) years; type of stroke: 44 cases of ischemic stroke and 16 cases of hemorrhagic stroke; part of hemiplegia: 34 cases of left hemiplegia and 26 cases of right hemiplegia. Observation group included 36 males and 24 females, with age 52–76 years and average age (65.08 ± 3.47) years; type of stroke: 43 cases of ischemic stroke and 17 cases of hemorrhagic stroke; part of hemiplegia: 32 cases of left hemiplegia and 28 cases of right hemiplegia. The baseline information of the two groups was homogenous (*P*˂0.05).

Inclusion criteria were as follows: ① patients who met the diagnostic criteria for stroke [7]; ② patients with no mental illness, with clear consciousness, and who can cooperate with the study; ③ the patient's information was true and complete. Exclusion criteria were as follows: ① patients with brain space-occupying disease; ② patients with severe heart, liver, kidney, and other organ diseases; ③ patients who were unconscious. According to the random number table method, the patients were divided into control group (60 cases) and observation group (60 cases).

### 2.2. Methods

#### 2.2.1. The Control Group Was Given Routine Care, including Routine Health Education, Monitoring of Blood Pressure, Heart Rate, and Other Vital Signs, and Patient's Condition Observation

1. Psychological intervention: actively communicate with patients and instruct correct stroke health knowledge to patients, correct patients' misunderstandings about the disease and eliminate patients' fear of disease through psychological counseling, and establish confrontation confidence in the disease and, at the same time, instruct family members to strengthen communication with patients, so that family members can care about and accompany patients more and jointly help patients get rid of negative emotions.
2. Health education: explain to patients the etiology, principles, treatment methods, preventive measures, rehabilitation training methods, etc., of stroke, so that patients fully understand stroke. Describe the significance of patting the patient's back for sputum removal and preventing bedsores, and let the family members be aware of the importance of patting the patient's back and regularly helping the patient to turn over and change positions. Illustrate the importance of early rehabilitation training for later prognosis, and encourage patients to actively cooperate with rehabilitation training.
3. Medication guidance: instruct patients to use medications on time, rationally and correctly. According to the patient's physical condition, dialectical treatment is adopted to reach yin and yang balance.
4. Acupuncture and massage: regularly relax the tendons, activate the collaterals, and stimulate righteousness.
5. Rehabilitation training: when the patient's condition is stable, take rehabilitation nursing training and guide the patient to perform rehabilitation exercises, including finger-pointing method, which can improve the patient's limb function and accelerate the patient's recovery. The points such as Jianpi, Quchi, and Hegu points in the upper limb and the Zusanli and Taixi points in lower limbs are pressed 3 times a day, 5 min each time.

#### 2.2.2. Observation Group Received Rehabilitation Nursing Based on the Theory of Interactive Standard

1. Preliminary preparation: ① *Training*. Nursing staff receive training based on the theory of interactive standard, fully master its methods, and can only take up their posts after passing the assessment. ② *Evaluation*. The staff communicate with the patient or family member immediately after admission, and collect the patient's clinical data, including the patient's condition and psychological status. Evaluate the patient's personal, interpersonal, and social systems to help patients understand themselves and discover their own problems. ③ *Diagnosis*. Make the corresponding nursing diagnosis according to the specific situation of the patient. ④ *Plan*. Combined with the patient's problems, the patient's information is comprehensively analyzed, and with the help of the rehabilitation therapist, the rehabilitation care goal sheet and plan are formulated together with the patient and family members.
2. Implementation of interactive rehabilitation nursing care: ① *Extreme Function Rehabilitation Training*. For patients with autonomous movement ability, they can be instructed to perform regular turning over, handshake training, and legs and feet stretch; for patients without autonomous movement ability, their family members can perform massage for the patient's paralyzed limbs to assist patients in moving their limbs. Attach importance to music sports therapy, and perform sports rehabilitation training in a cheerful and relaxing music environment to improve the patient's mental state and improve the quality of movement completion. ② *Daily Life Training.* Instruct patients to put on and take off clothes, wash daily, use tableware, perform hygiene management, carry out toilet management, perform walking exercises, etc. Train once a day, 30 min each time, and continue for 1 month. During the rehabilitation training period, the importance and necessity of rehabilitation training should be emphasized to enhance subjective initiative of the patients and the self-management ability. At the same time, notify family members to reduce the assistance to patients to reduce their dependence and improve the independent living ability of patients. ③ *Language Recovery Training*. For patients who are able to pronounce, instruct them to practice pronunciation and speak loudly during practice; for patients who are unable to pronounce, the responsible nurse can communicate with the patient through gestures, pictures, videos, etc., and the tone should be soft and clear when communicating. Explain to the patient how to train the oral muscles. Train the oral muscles by puffing your cheeks. Appropriate encouragement is given during the process to improve the patient's confidence in recovery. ④ *Psychological Rehabilitation Nursing*. According to the patient's psychological condition and other information learned before, the patient can receive attentive care and support from family, friends, and society. Instruct patients to carry out psychological suggestion, relieve bad emotions, and establish rehabilitation confidence to improve patients' compliance with rehabilitation training.

### 2.3. Observation Indicators

#### 2.3.1. Ability of Daily Living Activities

The Barthel Index Rating Scale was used to evaluate the ability of daily living activities of the two groups of patients before treatment and 7 days after treatment. A total of 10 items were evaluated, with a full score of 0–100 points. Evaluation criteria were as follows: ① 0 points, unable to meet the project standard; ② 1–19 points, assistance is completely needed in daily life; ③ 20–39 points, most of the assistance is needed in daily life; ④ 40–59 points, small part of assistance is needed in daily life; ⑤ 60–100 points, basically able to take care of themselves. The higher the score, the stronger the ability of daily living activities and the smaller the dependence [8].

#### 2.3.2. Degree of Neurological Deficit

The degree of neurological impairment was assessed by the National Institutes of Health Stroke Scale (NIHSS), with a score of 0 to 45 points. Evaluation criteria were as follows: ① 0–15 points, mild defects; ② 16–30 points, moderate defects; ③ 31–45 points, severe defects. The lower the score, the less impaired the patient's neurological function and the better the recovery [9].

#### 2.3.3. Quality of Life

The quality of life of patients was evaluated according to the Stroke Specific Quality of Life Scale (SS-QOL). The scale includes 12 dimensions (language, physical fitness, mood, personality, self-care, thought, vision, family role, social role, upper limb function, activity ability, and work ability), totaling 78 items. The total score is 0 to 248 points; the higher the total score, the higher the patient's quality of life [10].

#### 2.3.4. Compliance Rate

The rehabilitation standard rate was evaluated according to the patient's rehabilitation standard status. The standard rate = the number of standard/the total number of rehabilitation nursing cases × 100%.

#### 2.3.5. Nursing Satisfaction

Our hospital's self-made stroke patient nursing satisfaction survey questionnaire was used to investigate the nursing satisfaction of the two groups of patients, categorizing into very satisfied, satisfied, and dissatisfied classes. Satisfaction = (very satisfied + satisfied) number of cases/total number of cases × 100%.

#### 2.3.6. Time to Improved Limb Function and Rehabilitation Exercise Compliance

The time to improved limb function was recorded. The compliance assessment scale of rehabilitation exercise for patients with stroke was used to evaluate the compliance of the two groups of patients with rehabilitation exercise. The total score is 0–100 points; the higher the score, the better the compliance.

### 2.4. Statistical Analysis

The statistical analysis was done using SPSS24.0 statistical software. The measurement data were expressed as mean ± standard deviation, and the two independent samples *t*-test was performed to determine the statistical differences between groups. The paired samples *t*-test was performed to determine the statistical differences at different time points in one group; the count data were given as *n* (%), and *χ*2 test was performed to analyze the comparison. A level of statistical significance of *P* < 0.05 was accepted.

## 3. Results

### 3.1. Comparison of the Ability of Daily Living Activities

As shown in Table 1, before intervention, we observed no significant difference in the Barthel Index between the two groups (*P* > 0.05); after intervention, the Barthel Index of the two groups increased and the Barthel Index of the observation group was comparatively higher (*P* < 0.05). Interactive rehabilitation can continue to guide patients to carry out reasonable rehabilitation training while regularly adjust the rehabilitation plan according to the actual situation of patients, thus achieving higher ability of daily living activities.

### 3.2. Comparison of Neurological Deficits

The NIHSS scores of the two groups of patients were not significantly different before intervention (*P* > 0.05); after intervention, the NIHSS scores of the two groups of patients reduced and the NIHSS scores of the observation group were significantly lower than those of the control group (*P* < 0.05) (Table 2). NIHSS is an important scale for rapid assessment of stroke patients, which can assess and dynamically observe score changes. The result indicated the observation group achieved better neurological function after treatment.

### 3.3. Comparison of the Quality of Life

Before intervention, there was no significant difference in the SS-QOL scores of the two groups of patients (*P* > 0.05); after intervention, the SS-QOL scores of the two groups of patients improved and the increase of SS-QOL scores in the observation group was found to be significantly higher than those of the control group (*P* < 0.05) (Table 3). The ultimate goal of rehabilitation is to improve the quality of life; the result showed a better quality of life in the observation group.

### 3.4. Comparison of the Compliance Rate

**Figure 1:**
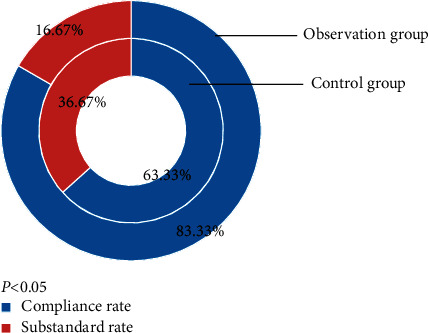
Comparison of compliance rates between the two groups of patients.

Figure 1 displays that the compliance rate was found to be in favor of the observation group (83.33 (50/60) vs 63.33 (38/60)) (*χ*^2^ = 6.136, *P*˂0.05). This is presumably because the interactive rehabilitation care improves the ability of daily living activities, neurological function, and quality of life to further increase the rehabilitation rate of patients.

### 3.5. Comparison of Nursing Satisfaction

**Figure 2:**
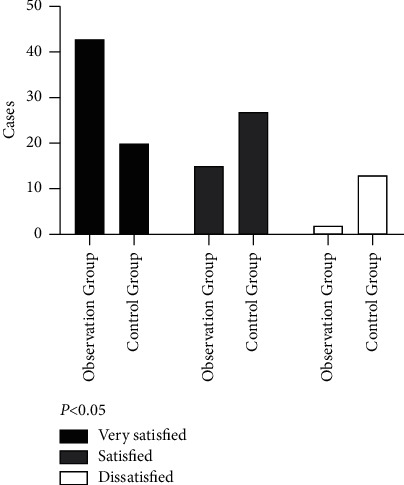
Comparison of nursing satisfaction between the two groups of patients.

The results showed that 43 cases were very satisfied in the observation group, 15 cases were satisfied, and 2 cases were dissatisfied; in the control group, 20 cases were very satisfied, 27 cases were satisfied, and 13 cases were dissatisfied. The total satisfaction of nursing care of patients in the observation group was superior to that in the control group (96.67% vs 78.33%) (*χ*^2^ = 9.219, *P˂*0.05) (Figure 2). It indicates that the interactive rehabilitation care outperformed conventional nursing in terms of nursing satisfaction.

### 3.6. Comparison of the Improvement Time of Limb Function

Compared with the control group, the limb function improvement time of the observation group was significantly shorter (*P* < 0.05) (Table 4). The recovery of limb function of patients after stroke is the fastest within 3 months; the results indicated that the function of interactive rehabilitation care quickly appears with the prolongation of time.

### 3.7. Comparison of Compliance with Rehabilitation Exercise

Table 5 lists that compared with the control group, the observation group had significantly higher rehabilitation exercise compliance scores (*P* < 0.05). It indicated that rehabilitation care based on the theory of interactive standards can improve the compliance of stroke patients with rehabilitation exercise.

## 4. Discussion

Over the past decades, the theory of interactive standards in clinical care has gradually been recognized by both doctors and patients. Research has found that rehabilitation care based on this theory is conducive to clarifying their rehabilitation goals, improving the subjective initiative of stroke patients, and promoting the patient's prognosis [11,12]. Interactive rehabilitation care could improve the ability of daily living activities, neurological function, and quality of life to further increase the rehabilitation rate of patients. The possible reason is that it emphasizes the participation of nursing staff and patients in the rehabilitation nursing process, which has switched from the conventional nursing staff-dominated nursing model to a model with patients involved [13], wherein the patients, like the responsible nurse, can also have their own rehabilitation care goal list [14]. Consequently, both nursing staff and patients have a clear understanding of their nursing or rehabilitation goals. In this regard, both parties can work together to combat diseases and improve the subjective initiative of patients [15].

The patient's nursing satisfaction is the standard for evaluating the quality of nursing, and the interactive standard theory is the best interpretation of “patient-centered.” It pays attention to the patient's participation and interaction and jointly formulates the rehabilitation goal list with the nursing staff, wherein their personality is respected, needs are met, quality of care is improved, and satisfaction with nursing work thus is boosted [16]. In interactive rehabilitation care, the nursing staff and the patient jointly set the rehabilitation goal and the communication between the two parties is strengthened, which is conducive to directly correcting the patient's cognitive errors and adjusting the patient's bad behavior. In addition, it is beneficial to improve patient's subjective initiative, enhance patients' self-management awareness and self-care ability, promote body function recovery, and speed up the recovery process [17]. Subsequently, cooperation degree is improved, and their compliance with rehabilitation exercises is thus correspondingly improved [18].

Unfortunately, the interactive standard rehabilitation nursing model also has certain shortcomings. This nursing model has higher requirements for nursing staff and patients than conventional nursing. Nursing staff have to receive rigorous training and pass the assessment before taking up the job. Importantly, patients should be conscious and clear-headed, have no communication barriers, and have relatively high compliance requirements [19,20]. Therefore, in consideration of actual situation of the patient, the nursing strategy should be correspondingly selected.

In summary, our study sheds light on improving stroke patients' neurological and limb functions and their daily life ability, quality of life, and nursing satisfaction by applying rehabilitation nursing based on the theory of interactive standards.

## Figures and Tables

**Table 1:** Comparison of the Barthel Index between two groups of patients ($\bar{x}$ ± *s*, points).

Groups	*n*	Before intervention	After intervention	*t*	*P*
Observation group	60	21.89 ± 3.46	45.08 ± 5.24	28.610	˂0.001
Control group	60	22.13 ± 3.25	37.56 ± 4.71	20.890	˂0.001
*t*		0.392	8.267		
*P*		0.696	0.001		

**Table 2:** Comparison of NIHSS scores between the two groups ($\bar{x}$ ± *s*, points).

Groups	*n*	Before intervention	After intervention	*t*	*P*
Observation group	60	27.64 ± 4.52	10.15 ± 3.01	24.950	0.001
Control group	60	26.93 ± 4.79	13.47 ± 3.45	17.660	＜0.001
*t*		0.835	5.617		
*P*		0.405	˂0.001		

**Table 3:** Comparison of SS-QOL scores between the two groups ($\bar{x}$ ± *s*, points).

Groups	*n*	Before intervention	After intervention	*t*	*P*
Observation group	60	98.73 ± 6.49	196.85 ± 8.31	72.080	˂0.001
Control group	60	99.14 ± 6.58	127.42 ± 7.15	22.540	˂0.001
*t*		0.344	49.060		
*P*		0.732	˂0.001		

**Table 4:** Comparison of improvement time of limb function between the two groups ($\bar{x}$ ± *s*, d).

Groups	*n*	Improvement time of limb function
Observation group	60	13.09 ± 3.26
Control group	60	19.72 ± 4.80
*t*		8.851
*P*		˂0.001

**Table 5:** Comparison of rehabilitation exercise compliance scores between the two groups ($\bar{x}$ ± *s*, points).

Groups	*n*	Rehabilitation exercise compliance scores
Observation group	60	82.17 ± 4.59
Control group	60	91.46 ± 5.01
*t*		10.590
*P*		<0.001

## Data Availability

The data used to support the findings of this study are included within the article.
